# Primary Palmar Hyperhidrosis (PPH) Accompanied With Nevus Flammeus: A Case Report

**DOI:** 10.7759/cureus.38723

**Published:** 2023-05-08

**Authors:** Alen Sam Saji, Aliza Paudyal, Veylenta Audry De Souza, Sandeep Soman Pillai Radhamoney Amma, Nitya Prajwalita Rai

**Affiliations:** 1 Internal Medicine, West China Hospital, West China Medical School, Sichuan University, Chengdu, CHN; 2 Dermatology, B.P. Koirala Institute of Health Sciences, Dharan, NPL; 3 Dermatology, West China Hospital, West China Medical School, Sichuan University, Chengdu, CHN

**Keywords:** chatgpt aided case report, nevus flammeus, palmar hyperhidrosis, hyperhidrosis, dermatology

## Abstract

Palmar hyperhidrosis (PH) is a medical condition characterized by excessive sweating in the palms of the hands, which can result in significant distress and impairment in daily activities. Flammeus nevus, on the other hand, is a benign vascular lesion that appears as a red or purplish discoloration on the skin, commonly found on the face, neck, or trunk. In some cases, flammeus nevus can co-occur with PH, leading to increased sweating in the affected area. This condition can cause significant psychosocial impact, affecting an individual's quality of life (QoL) and self-esteem. We present a case report of a patient presenting with PPH with flammeus nevus. There is currently limited information available on the relationship between PH and flammeus nevus, and more research is needed to better understand this phenomenon; here we have reported the presentation of a patient. In conclusion, PH accompanied with flammeus nevus is a condition that requires prompt attention and management to mitigate its adverse effects. We have used ChatGPT to aid in structuring and writing this case report.

## Introduction

Primary palmar hyperhidrosis (PPH) is a pathological condition that involves excessive sweating of palms beyond physiological needs due to the over secretion of eccrine glands. The main cause of PPH is proposed to be due to overstimulation of the sympathetic nervous system which controls the body's "fight or flight" response [1]. Patients suffering from this condition usually experience social or emotional embarrassment along with other psychological problems. Other causes of the condition can also be due to genetic factors. PPH can be triggered by physical activities or stressful situations or other miscellaneous factors. Symptoms of PPH are cold or/and wet palms, pale or blue-ish palms or even swelling of the fingers [2]. Palmar hyperhidrosis (PH) can also be secondary to other medical conditions, such as hyperthyroidism or Parkinson's disease [3]. The symptoms of PH can range from mild to severe. In mild cases, the excessive sweating is limited to the palms of the hands and may only occur in certain situations, such as during physical activity or in response to stress. In more severe cases, the excessive sweating can occur constantly and interfere with daily activities. The excessive sweating can also lead to secondary skin conditions, such as fungal or bacterial infections, or chafing of the skin [4]. There has been no reported co-relation between PH and flammeus nevus, however, we report the possibility of a psychological factor that may increase the patient discomfort and/or affect the patient's QoL. We had used ChatGPT to aid in writing this case report.

## Case presentation

**Figure 1:**
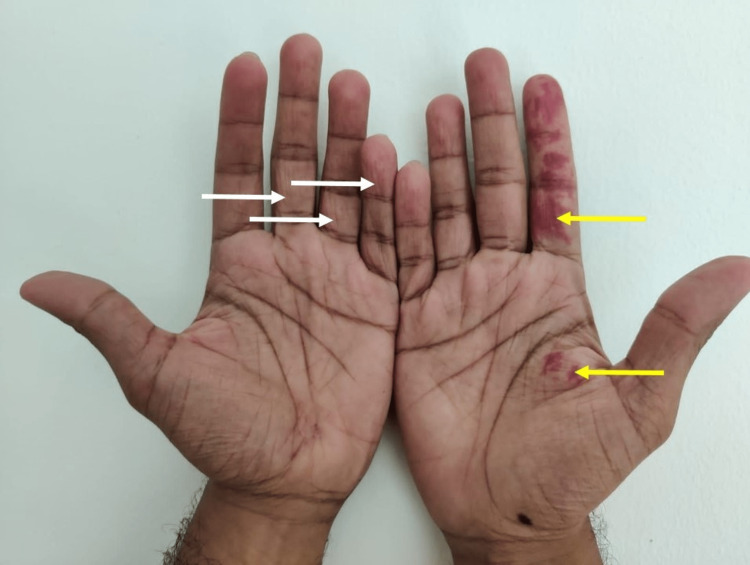
Unilateral nevus flammeus pigmentation on the right hand (yellow arrows). Hand wrinkling caused by prolonged hyperhidrosis (white arrows).

The patient is a 23-year-old apparently healthy male with PH and port wine stains. The patient reports excessive sweating in his hands and presence of red pigmented marks on his palms. The patient has been experiencing excessive sweating in his hands from childhood. This sweating has caused significant distress in his daily activities and has had a significant impact on his personal and professional life. The patient also reports the presence of pigmented marks on his hands that have been present since birth. These birthmarks are known as port wine stains or flammeus nevus. They are flat, reddish marks that varied in size and were mainly present on the patient's second digit of the right hand and proximal to the right thumb (Figure 1). The patient reported excessive sweating on the hand with the birthmark and also reports a history of stress related to his college studies; he had also reported to have smoking and occasional drinking history. 

Upon physical examination, the patient's hands were noted to have bilateral excessive sweating, with dampness and a mild odor. The port wine stains on his hands were observed to be of various sizes and were noted to be flat and red in color. These birthmarks can range in size and can be unsightly, causing emotional distress for some patients [5].

Firstly, secondary hyperhidrosis was excluded using the patient history and after correlation of his clinical history with palmar lesion morphology, the diagnosis of PH and Port Wine Stains was established. The hyperhidrosis disease severity scale (HHDS) was used to determine the severity of the disease and the patient scored a three on a four-point scale (Table 1). 

**Table 1:** HHDS assessment for the patients. HHDS, hyperhidrosis disease severity scale

Score	HHDS [6]
1	Sweating is never noticeable and never interferes with my daily activities
2	Sweating is tolerable but sometimes interferes with my daily activities
3	Sweating is barely tolerable and frequently interferes with my daily activities
4	Sweating is intolerable and always interferes with my daily activities

The patient was recommended a combination of treatments, including antiperspirants, iontophoresis, botulinum toxin injections but due to personal reasons was only willing to use topical antiperspirants like aluminum chloride. The patient was prescribed topical aluminum chloride to be used one time a day for two to three times a week as necessary. 

In addition, the patient was advised to manage his stress levels, stop alcohol consumption, and quit smoking. Stress management techniques, such as exercise, meditation, and therapy, were recommended to aid the patient with coping with stress and reducing the impact it has on his condition.

After two months, during the follow-up the patient reported a significant reduction in excessive sweating in his hands and reported a score of two according to HHDS. The patient did not report any adverse effects. He also reported improved self-esteem and better mood as a result of the improvement in his condition.

## Discussion

Primary palmar hyperhidrosis is a type of localized hyperhidrosis that falls under the umbrella of somatic disorders and has various triggers. Treatment modalities for PPH are mostly aimed at treating the excessive palmar perspiration that patients experience. The mechanism behind PPH is still not fully elucidated. Flammeus nevus are vascular malformations that are formed due to the abnormal malformations of blood vessels in the skin. We did not find any co-relation between the two conditions in the patient but there may be a psychological factor that may play a role in the increased discomfort of the patient as the patient reported to have excessive sweating on the right hand (with nevus flammeus) compared to the left but in physical examination the hyperhidrosis seemed to be bilaterally symmetrical and there was no found association of PPH with nevus flammeus.

In an epidemiological survey conducted among young patients in China in 2007 by Xu et al. [7], the prevalence of PPH was only 2.08%, but more recent estimates of PPH are 2%-5 % and were found to affect male and female sexes equally. Some of the most commonly used medical therapeutic modalities for PPH include:

*Topical antiperspirants*: Topical aluminium chloride (20%) is generally the first-line treatment option in most cases of mild to moderate severity of PPH. The aluminum salts act as an obstructive plug in the eccrine glands and reduces their secretions by causing damage to the secretory cells. However, the plug formation has been found to increase the hydrochloric acid secretions that can cause skin irritations [8]. A study by Yanagishita et al. [9] has shown low risk of systemic damage caused by aluminum absorption and accumulation in visceral organs.

*Iontophoresis*: It is one of the first-line treatment choices for PH and has a long-term history of safe usage. It works by passing an ionized substance through the skin surface by making use of direct electrical current. Even though the mechanism of action is not very clear it is theorized to work by plugging of eccrine glands with ions [10]. It is regarded to be the simplest, safest, and most cost-effective treatment option for PH. The side effects of iontophoresis are typically minor and do not indicate cessation of the therapy. They can be avoided with the use of proper technique and patient education [10-11].

*Botulinum toxin (BTX)*: Even though BTX is one of the deadliest known poisons, it has several medical applications when used in small doses, such as treating PH. It is generally a second-line treatment for PPH after the failure of topical treatments. It works by blocking the cholinergic innervation of the eccrine glands [12-13]. It is well tolerated and safe to use when done using a proper technique by an experienced drug administrator. It is contraindicated in pregnant and breastfeeding women.

*Anticholinergics*: These are useful adjuncts given with other medications when other treatment options are not successful in improving the patient’s condition [14] and they work by acting as an antagonist in muscarinic receptors [15]. The only Food and Drug Administration (FDA) approved agent to date is glycopyrrolate [16] but it is often reported to have side effects like dry mouth that may lead to treatment cessation [17-18].

*Photodynamic therapy (PDT)*: This treatment was first reported in 2014 by Mordon et al. [19] and was confined to treating axillary hyperhidrosis but many recent studies has shown the effectiveness of PDT in the treatment of PPH [20-22]. It works by using a photosensitizer and visible light with oxygen to create reactive oxygen species that will cause cell organelle damage or cell death that would in turn stop excessive production of sweat from the eccrine glands [20]. The results are reported to be maintained up to 3 months [21] but larger clinical studies with longer follow up is required to validate the conclusions.

There are other reported methods such as hypnosis, psychotherapy [23-24], etc. that have also been reported as adjunct to first-line medications, as in our case we have recommended breathing exercises and meditation to decrease the patients’ stress levels in addition to physical exercise, stopping alcohol consumption, and smoking cessation. In our case, we have found that with moderate severity of PH, the patient was still able to get significantly improved quality of life (QoL) by just using topical aluminum chloride solution and making extensive behavioral changes.

As a language model that was created by OpenAI, ChatGPT has access to an extensive database of information and was able to generate high quality content quickly and efficiently. Although not every information provided by ChatGPT would be necessarily correct, it was able to assist with tasks such as topic generation, topic outlining, grammar check, paraphrasing, etc. Its human-like language ensures coherent and grammatically correct sentences. Incorporating AI tools like ChatGPT can improve the quality of article writing and also save authors a lot of time. However, the limitations of AI in writing that includes inability to understand context unless specified, overreliance of source materials which could affect the accuracy and reliability of information, limited creativity, etc. should be acknowledged.

## Conclusions

We have reported a rare occurrence of PH with nevus flammeus and more research and investigations are required to conclude any associations between the two. The patient did not choose treatment methods like iontophoresis or BTX injections to treat his condition even though he got a score of three in HHDS. The patient has reported significant improvement in his QoL after 2 months of topical antiperspirant use. We had discussed about the existing treatment options in the treatment of PPH. Although AI has a great potential to be an aid in academic writing, it has limitations that must be acknowledged. Consequently, information generated by AI should be fact-checked for accuracy and credibility. 

## Figures and Tables

**Figure 2:**
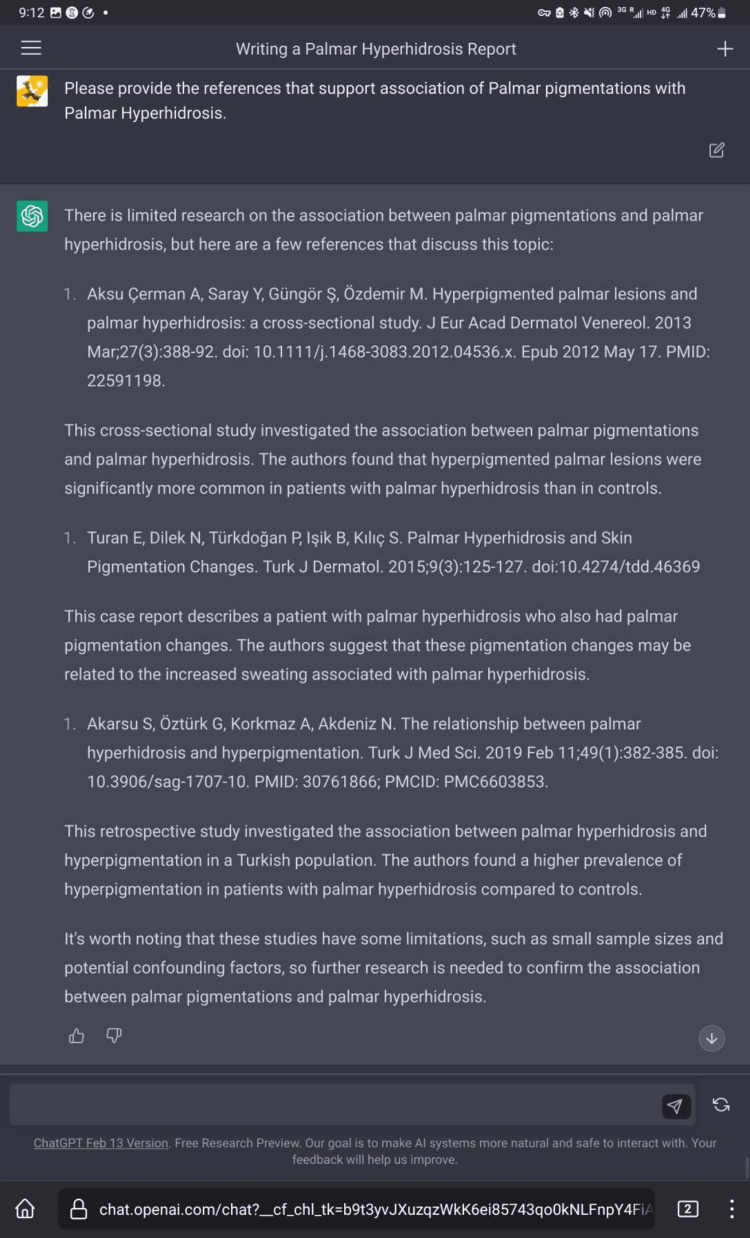
Non-existent references provided by the AI. AI, artificial intelligence

**Figure 3:**
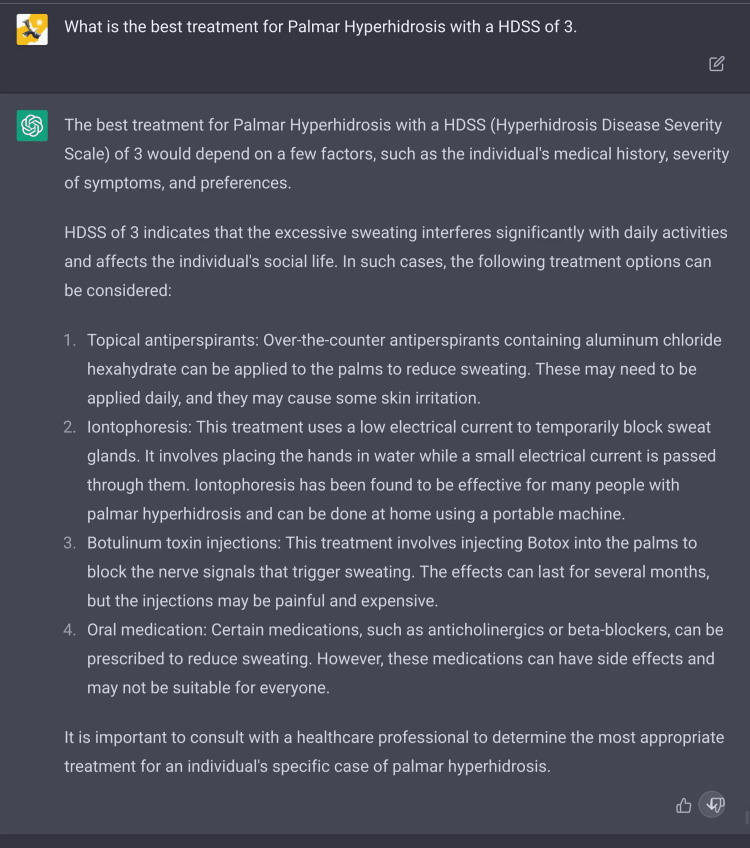
Chat Prompt: 'What is the best treatment for Palmar hyperhidrosis?'

## Appendices

We have also noticed that ChatGPT can create references that does not exist to support a statement proposed by the user. This is a limitation that could possibly affect academic writing using AI. Besides this, there are other limitations of AI in academic writing that includes inability to understand context unless specified, overreliance of source materials which could affect the accuracy and reliability of information, limited creativity, etc. (Figure 2).

We verified that the references provided does not exist by doing a detailed search of the literature on major databases like PUBMED, Cochrane, Google scholar etc. We observed that ChatGPT's generated recommendations on therapeutics closely resembled the initial advice given to the patient by the physician (Figure 3). 

We have also noticed that unless the Chat Prompts are very specific, the generated information could be repetitive. 
